# Physiological and transcriptome analysis reveals the differences in nitrate content between lamina and midrib of flue-cured tobacco

**DOI:** 10.1038/s41598-022-07011-y

**Published:** 2022-02-21

**Authors:** Yuqing Feng, Yuanyuan Zhao, Yafei Li, Jun Zhou, Yujing Li, Hongzhi Shi

**Affiliations:** 1 National Tobacco Cultivation & Physiology & Biochemistry Research Center, Tobacco Harm Reduction Research Center of China Tobacco, Henan Agricultural University, Zhengzhou, 450002 China; 2 Beijing Cigarette Factory, Shanghai Tobacco Group Co., Ltd., Beijing, 100024 China

**Keywords:** Photosynthesis, Carbohydrates

## Abstract

Nitrate is an important precursor of tobacco-specific nitrosamines (TSNAs) and a remarkable difference in nitrate accumulation between lamina and midrib of flue-cured tobacco has long been observed. However, the physiological and molecular mechanisms underpinning this difference remain poorly understood. In this study, physiological and genetic factors impacting nitrate accumulation were identified in pot experiments using flue-cured tobacco K326 with contrasting nitrate content between lamina and midrib. The results showed that three times higher NO_3_-N content was observed in midrib than that in the lamina, along with lower pigment, NH_4_-N content, nitrate reductase activity (NRA), sucrose synthetase activity (SSA), and glutamine synthetase activity (GSA) in midrib. Transcriptome analysis revealed that expression of genes involved in porphyrin and chlorophyll metabolism, carotenoid biosynthesis, photosynthesis-antenna proteins, photosynthesis, carbon fixation in photosynthetic organisms, starch and sucrose metabolism, nitrogen metabolism, and biosynthesis of amino acids were significantly lower in midrib than in lamina. qRT-PCR results showed that the expression level of nitrate transporter genes *LOC107782967*, *LOC107806749*, *LOC107775674*, *LOC107829632*, *LOC107799198*, *LOC107768465* decreased by 2.74, 1.81, 49.5, 3.5, 2.64 and 2.96-folds while *LOC107789301* increased by 8.23-folds in midrib but not in lamina. Reduced chlorophyll content might result in low carbohydrate formation which is the source of energy and carbon skeleton supply, then the low capacity of nitrogen reduction, assimilation and transportation, and the poor ability of nitrate reallocation but the high capacity of accumulation might lead to nitrate accumulation in midrib. The results laid the foundation for reducing nitrate content and TSNA formation in tobacco midribs and their products.

## Introduction

Tobacco is an industrial crop that is widely grown throughout the world. Tobacco leaf consists of lamina and midrib, with midrib accounting for about 25–30% of the leaf weight. Not only lamina but also midrib is widely used as raw materials for cigarette production through the making of reconstituted tobacco sheets or midrib cut. Therefore, tobacco midribs have great value when scientifically processed. Midrib has a lower tar level, so it plays a significant part in reducing the hazards of cigarettes^1^. The usage of midrib is also beneficial to cost cutting, thus improving the utilization efficiency of tobacco raw materials. However, the disadvantage of midrib is also obvious, among which is substantial higher levels of nitrate content and subsequent higher formation and accumulation of tobacco-specific nitrosamines (TSNAs)^2,3^ than that in the lamina. Nitrate content in midrib of cured tobacco leaf is usually more than 10 times higher than that in the lamina of the same cured leaf^2^.

TSNA is prone to induce malignant tumors in animals and was classified as the first class carcinogen by the International Agency for Research on Cancer^4^. It is well recognized that nitrate is an important precursor of tobacco-specific nitrosamines (TSNAs). Nitrate may easily be reduced to nitrite by microbial activity during leaf curing^5^ or produce gaseous NOx during leaf storage under warm or hot conditions^6^, and the subsequent nitrosation of tobacco alkaloids by these nitrosating agents may lead to much increased levels of TSNA formation and accumulation in midrib^1^. Therefore, the reduction of nitrate content is a key for reducing TSNA formation, and the investigation of the mechanisms of nitrate accumulation in midrib is essential, so as to lay the foundation for reducing nitrate content and TSNA formation in tobacco midribs and their products.

Nitrate (NO_3_^−^) is one of the main sources of nitrogen absorption by plants, which will accumulate to a large extent in plant cell vacuoles if not being reduced, reused, or transported into the cytoplasm^7^. Once absorbed by root cells, a larger proportion is transferred to the shoot, where it is rapidly turned into nitrite by nitrate reductase (NR) and nitrite reductase (NiR), and subsequently incorporated into glutamine by glutamine synthetase (GS), which is metabolized to glutamate (Glu) and glutamine (Gln) by Gln synthetase (GS) and Glu synthase (GOGAT), respectively^8,9^.

Carbon metabolism is highly correlated with nitrogen metabolism in plants. N assimilation requires both energy and organic carbon (C) which are provided by photosynthesis^8,10^. A previous study demonstrated that the lowering of pigment content, carbon fixation, and nitrogen assimilation were the main causes of nitrate accumulation in burley tobacco^11^. Moreover, some genes and transcription factors involved in nitrate transport, signaling, and use efficiency can affect the content of nitrate. Four protein families are known to be involved in nitrate uptake, distribution, or storage: the Nitrate Transporter 1/Peptide Transporter (NPF) family, the Nitrate Transporter 2 (NRT2) family, the Chloride Channel (CLC) family, and the Slow Anion Associated Channel Homolog (SLC/SLAH) family^9^. *AtNPF6.3* (also known as *CHLORATE RESISTANT 1*, *CHL1*, or *NRT1.1*) was the first dual-affinity nitrate transporter and also founctions as a nitrate sensor^12^. *OsNRT1.1B/OsNPF6.5* also operates as a dual-affinity nitrate transporter and mediates nitrate uptake and root-to-shoot transport^13^. *AtNPF7.3/NRT1.5* modulates xylem loading of nitrate in root pericycle cells^14,15^. *AtNPF7.2/NRT1.8* is chiefly expressed in xylem parenchyma cells and more nitrate is found in xylem sap in *npf7.2* mutants^14^. Interestingly, the functions of *NPF7.2* and *NPF7.3* are antagonistic, and expressions of *NPF7.2* and *NPF7.3* are inversely regulated upon stress treatments^13,16^. *AtNPF5.11*, *AtNPF5.12*, and *AtNPF5.16*, localized in tonoplast, were proposed to mediate nitrate efflux from vacuoles and to regulate nitrate distribution between roots and shoots^17^. Moreover, *AtNPF6.2/NRT1.4* is predominantly expressed in the petiole and midrib of leaves^18^. Compared with the wild type, less nitrate accumulates in the petiole, but more nitrate is detected in the leaf blade of *npf6.2* mutants, indicating that *NPF6.2* participates in nitrate storage of the petiole. NLPs have been suggested to be involved in mediating the early N response. For instance, transcription factor *NIN-LIKE PROTEIN 7* (*NLP7*) was identified as a primary regulator in nitrate response in Arabidopsis and regulates the expression of several nitrate-responsive genes including *NITRATE REDUCTASE 1* (*NIA1*), *NIA2*, *NRT2.1*, and *NRT2.2*^19,20^. Furthermore, *OsNLP4* transactivates the NRE motif at the promoter of *OsNiR* encoding nitrite reductase that is a key enzyme determining nitrogen assimilation in rice^21^. In addition to the genes above, other genes which mediate, for example, nitrate signalling and transcription factors play an essential role in nitrate metabolism^22,23^.

In recent year, the midrib is also widely used in cigarette production. However, the nitrate content of the midrib is markedly higher than that of the lamina. Shi et al.^11^ compared the carbohydrate and nitrate accumulation of flue-cured tobacco with that of burley tobacco, while inclusive studies about lamina and midrib have never been reported. This study aimed to identify the physiological and transcriptome differences between the lamina and midrib, so as to reveal the mechanism of nitrate accumulation in midrib. Significant findings were obtained that would provide insight into the difference in carbon and nitrogen metabolism and valuable gene resources that might explain the reason why midrib had higher nitrate content.

## Results

### Differences in enzymes activities and nitrogen compounds between lamina and midrib

**Figure 1:**
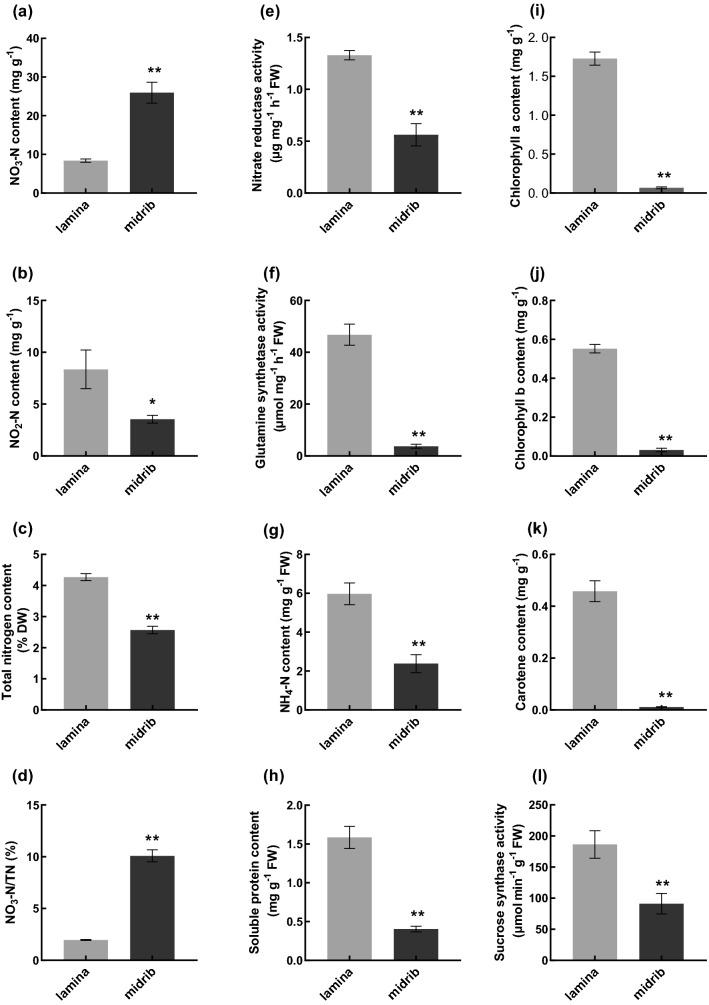
(**a**) NO_3_-N content; (**b**) NO_2_-N content; (**c**) total nitrogen content; (**d**) NO_3_-N/TN; (**e**) nitrate reductase activity; (**f**) glutamine synthetase activity; (**g**) NH_4_-N content; (**h**) soluble protein content; **(i)** chlorophyll a; (**j**) chlorophyll b; (**k**) carotene content; (**l**) sucrose synthetase. Symbols ** and *indicates that the significant differences between lamina and midrib at 0.01 and 0.05.

The results showed that pigment content, enzyme activities, and nitrogen compounds were different between lamina and midrib (Fig. 1a–l). Chlorophyll a content, chlorophyll b, and carotenoid contents were significantly lower in midrib than those in the lamina. Also, SSA was always lower in midrib than that in the lamina. Lower pigment content may have an influence on carbon fixation and lead to low carbohydrate accumulation in midrib. Also the nitrate reductase activity (NRA) and glutamine synthetase activity (GSA) were lower in midrib than in the lamina. In addition, NH_4_-N, NO_2_-N, total nitrogen content (TN), and soluble protein content in midrib were dramatically lower than those in midrib while the NO_3_-N content and the ratio of NO_3_-N/total nitrogen content (TN) were significantly higher, indicating that the ability of nitrate reduction and assimilation in lamina was higher than midrib. It is noteworthy that the NO_3_-N content accumulated to 25.96 mg g^−1^ in midrib and was 3.1 times than that in the lamina, which might be due to the weak ability of nitrogen reutilization, leading to nitrate accumulation in midrib.

### Quality control, gene expression, and correlation analysis between samples

**Figure 2:**
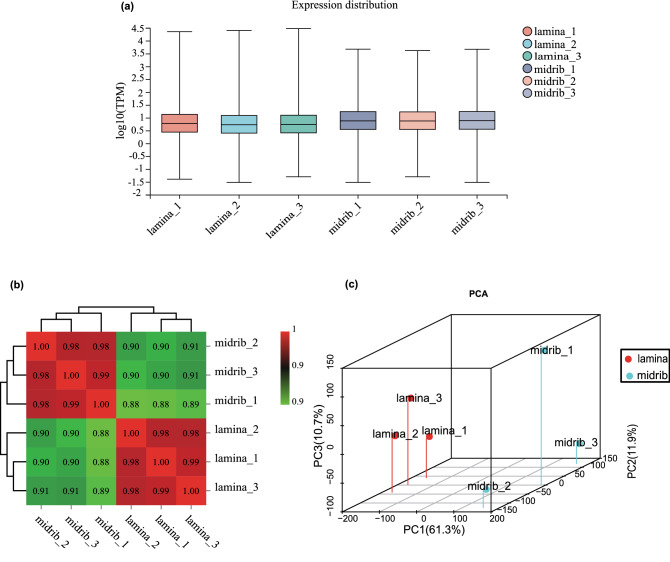
(**a**) Gene expression levels in samples. (**b**) Heatmap of correlation between samples. (**c**) The results of principal component analysis.

After filtering the raw reads, a high rate of clean reads from each sample was achieved. In short, the mapping rates of all the samples to the reference genome were above 93%, the GC content of all samples was stable with the distribution ranging from 43.16 to 44.09% and the QC30 value of all samples was above 91% (Table 1), implying successful library construction and RNA sequencing. As shown in Fig. 2a, the FPKM expression levels for each sample were calculated. In addition, the range of correlation coefficients among intra-class was distributed between 0.98 and 1.00 (Fig. 2b). And principal component analysis (PCA) of the data profiles from all 6 samples revealed a high correlation among all samples (Fig. 2c). These results demonstrated that the sequencing data in the present study were adequately representative and valid.

**Table 1:** The primers used in real-time PCR.

Primer name	Primer sequence (5′–3′)
LOC107782967-TKF	TCAGACATGGGTTCCGTGTG
LOC107782967-TKR	GGGGGTCAGCAACATAGCAA
LOC107806749-TKF	CAACACGACAGGCAAAGCAG
LOC107806749-TKR	CAAATCATCGGCAGCAGCAT
LOC107775674-TKF	TGGAGGGCTATGCCTTATGTT
LOC107775674-TKR	AAGCACCGAGCAATGGTATGA
LOC107829632-TKF	CAGTGGTCGTTGATGGTGATG
LOC107829632-TKR	TTGATAGGCTGGCAGGAGGTA
LOC107799198-TKF	GTTCCGATTTGTCGTCGTTTC
LOC107799198-TKR	GTGGCATTTGCATCATTGGTC
LOC107768465-TKF	GGATGAAGGAATGTGGGCTCT
LOC107768465-TKR	TCTTCGGTTTCTGGTGTCTCG
LOC107789301-TKF	TCCGTGCCAACGAACAAAT
LOC107789301-TKR	TCGACTGCAACGCCATCTT
LOC107770138-TKF	GGGTTGTCCATGTCTTCCTCA
LOC107770138-TKR	TCCAAGTGCCCGTCGTTTA
Actin-TKF	CTGAGGTCCTTTTCCAACCA
Actin-TKR	TACCCGGGAACATGGTAGAG

### Differentially expressed gene (DEG) selection, Gene Ontology (Go) enrichment, and Kyoto Encyclopedia of Genes and Genomes (KEGG) pathway analysis of DEGs

**Figure 3:**
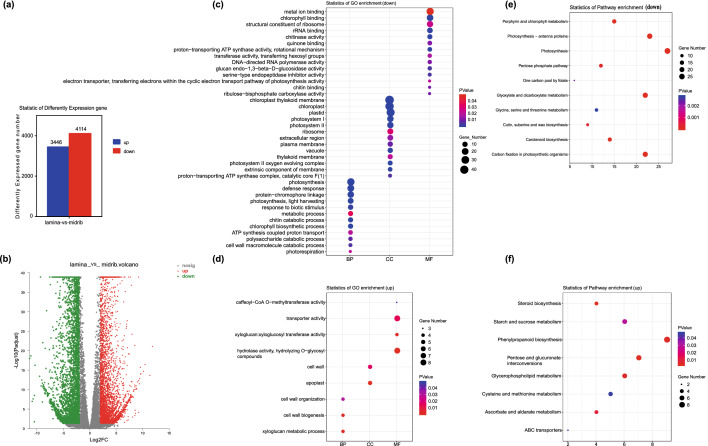
(**a**) The number of differentially expressed genes between lamina and midrib. (**b**) The volcano of differentially expressed genes between lamina and midrib. (**c,d**) Go enrichment of differentially expressed genes between lamina and midrib. (**c,d**) KEGG enrichment of differentially expressed genes between lamina and midrib.

The fold change (FC) > 2 or FC < 0.5, and a *P.adjust* < 0.05, were used thresholds to determine the DEGs. A total of 7560 DEGs (3446 upregulated and 4114 downregulated) were identified between the lamina and the midrib groups (Fig. 3a). And the volcano of differentially expressed genes between the lamina and the midrib was achieved (Fig. 3b).

The DEGs in lamina vs midrib were further analyzed using Gene Ontology (Go) enrichment, and Kyoto Encyclopedia of Genes and Genomes (KEGG) analyses (Fig. 3c–f). In detail, the down-regulated DEGs in lamina vs midrib were significantly enriched in photosynthesis-antenna proteins (ko00196), photosynthesis (ko00195), porphyrin and chlorophyll metabolism (ko00860), carbon fixation in photosynthetic organisms (ko00710), carotenoid biosynthesis (ko00906), photosystem II (GO:0009523), and photosystem I (GO:0009522). The up-regulated genes were mostly involved in cell wall organization (GO:0071555), phenylpropanoid biosynthesis (ko00940), steroid biosynthesis (ko00100), xyloglucan metabolic process (GO:0010411), pentose and glucuronate interconversions (ko00040), and transporter activity (GO:0005215).

### Comparative analysis of DEGs correlated with carbon and nitrogen metabolism

**Figure 4:**
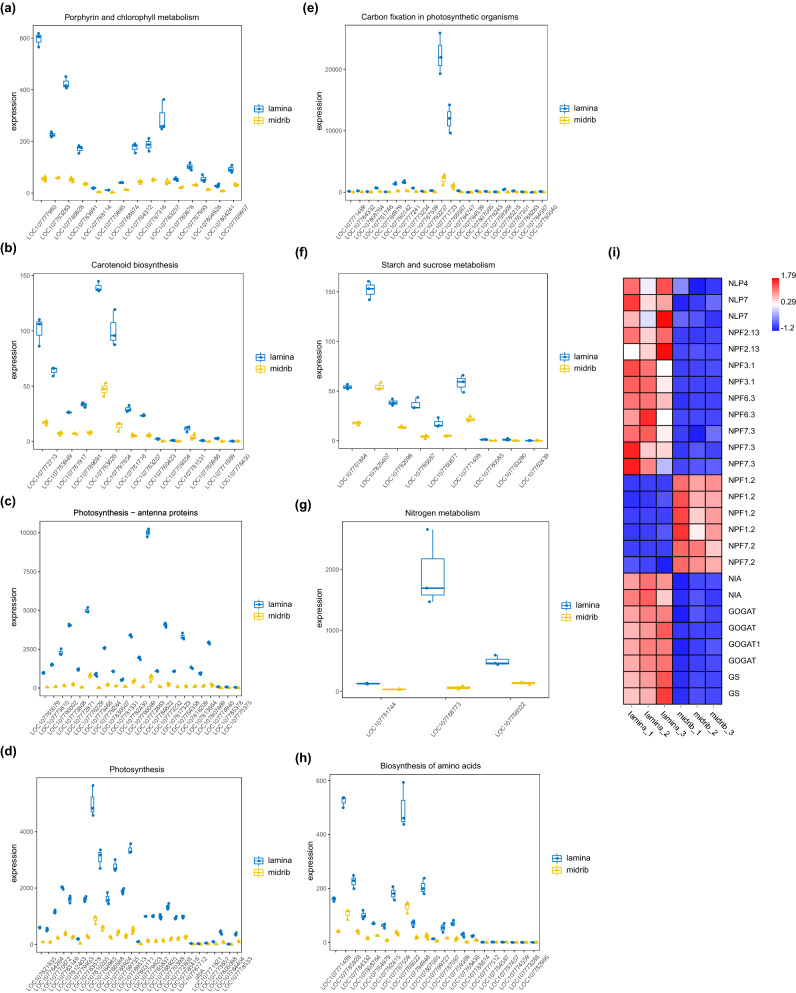
(**a**) Porphyrin and chlorophyll metabolism; (**b**) carotenoid biosynthesis; (**c**) photosynthesis-antenna proteins; (**d**) photosynthesis; (**e**) carbon fixation in photosynthetic organisms; (**f**) starch and sucrose metabolism; (**g**) nitrogen metabolism; (**h**) biosynthesis of amino acids; (**i**) expression of genes involved in nitrate response, transport and assimilation. Box-whisker plot represents dispersity of minimum, first quartile, median, third quartile in genes expression level of treatments. Y-axis represents expression level. The columns represent six samples. The name of gene is on the right side, the up-or down-regulated proteins are indicated in red and green, respectively. The intensity of the colors increases with increasing expression level as noted on the color bar on the right side.

Transcriptome sequencing technology provides a large amount of information regarding the DEGs that are involved in specific biological responses. Figure 4 showed that porphyrin and chlorophyll metabolism, carotenoid biosynthesis, photosynthesis-antenna proteins, photosynthesis, carbon fixation in photosynthetic organisms, starch and sucrose metabolism, nitrogen metabolism, and biosynthesis of amino acids were lower in midrib than in lamina. In addition, we searched the genes involved in porphyrin and chlorophyll metabolism (*LOC107777980*, *LOC107786828,* and *LOC107788874*) (Fig. 4a), carotenoid biosynthesis (*LOC107772713*, *LOC107763949*, *LOC107763628*, and (*LOC107797654*) (Fig. 4b), photosynthesis-antenna proteins (*LOC107773808*, *LOC107776229*, *LOC107778264*, *LOC107782430*, *LOC107772663*, *LOC107773232,* and *LOC107764358*) (Fig. 4c), photosynthesis (*LOC107763149*, *LOC107810205*, *LOC107784985*, *LOC107766588*, and *LOC107768924*) (Fig. 4d), carbon fixation in photosynthetic organisms (*LOC107780142*, *LOC107777241*, *LOC107771723*, and *LOC107766567*) (Fig. 4e), starch and sucrose metabolism (*LOC107761864*, *LOC107825407,* and *LOC107771409*) (Fig. 4f), nitrogen metabolism (*LOC107768773*, and *LOC107766022*) (Fig. 4g) and biosynthesis of amino acids (*LOC107785928*, *LOC107784332*, *LOC107766022*, and *LOC107794948*) (Fig. 4h) were greatly suppressed in midrib. To explore the reason why midrib holds higher nitrate than lamina, we analyzed the differences in gene expression levels of nitrate response, transport, and assimilition. The results showed that genes of *NLP4 (LOC107782967)*, *NLP7 (LOC107806749)*, *NPF2.13 (LOC107775674)*, *NPF3.1 (LOC107829632)*, *NPF6.3 (LOC107799198)*, *NPF7.3 (LOC107768465)*, *NIA (LOC107794079)*, *GS (LOC107802035)*, and *GOGAT (LOC107781744)* were down-regulated in midrib while genes of *NPF1.2 (LOC107789301)* and *NPF7.2 (LOC107770138)* were up-regulated in (Fig. 4i), which might also be the cause for higher nitrate content in the midrib.

### Expression levels of genes related to nitrate transport

**Figure 5:**
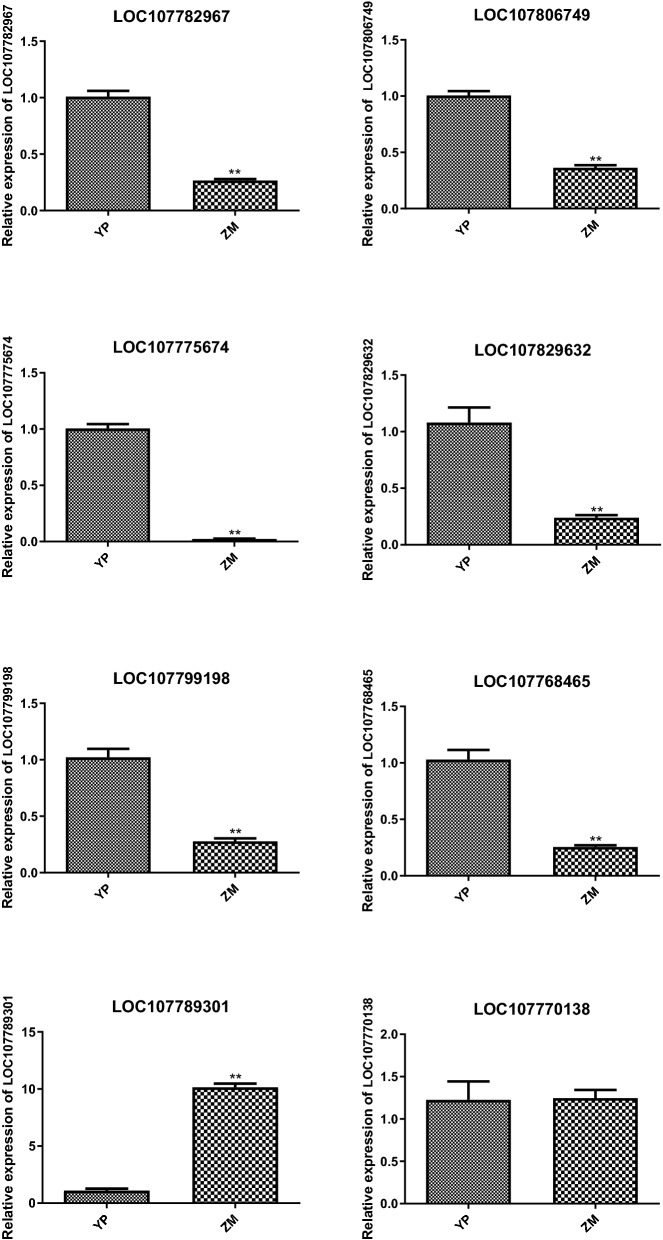
Expression levels of genes related to nitrate transport by qRT-PCR. The x-axis indicates the two samples. YP: lamina of flue-cured tobacco; ZM: midrib of flue-cured tobacco. The left y-axis indicated relative expression level of qRT-PCR. Error bars represent standard error of mean.

Nitrate transporters play an essential role in nitrogen metabolism. The expression level of genes involved in nitrate transporting (LOC107782967, LOC107806749, LOC107775674, LOC107829632, LOC107799198, LOC107768465) was down-regulated while LOC107789301 and LOC107770138 were up-regulated in midrib compared to that in the lamina (Fig. 5). And the qRT-PCR results showed that the expression patterns of the eight genes were identical to those detected by transcriptome sequencing, which confirmed the reliability of RNA-seq data and explained the reason why nitrate content was higher in midrib than that in the lamina.

## Discussion

In recent year, the midrib has been widely used in cigarette production in the form of tobacco sheets. However, our study showed that the midrib had higher NO_3_-N content of more than 3 times than lamina (Fig. 1a), which is not beneficial to tobacco safety and harm reduction. One strategy to decrease the content of nitrate is to identify the physiological and molecular mechanisms contributing to nitrate accumulation in the midrib. In the studies presented here, the pots experiment was employed to study the physiological and transcriptome differences between lamina and midrib. Overall, the present study demonstrated that the expression of genes involved in porphyrin and chlorophyll metabolism, carotenoid biosynthesis, photosynthesis-antenna proteins, photosynthesis, carbon fixation in photosynthetic organisms, starch and sucrose metabolism, nitrogen metabolism, and biosynthesis of amino acids were significantly lower in midrib than in the lamina (Fig. 4a–h), which might be the cause for higher nitrate accumulation in the midrib.

It has long been recognized that chlorophyll content is used as an indicator of photosynthetic capacity and photosynthesis and C metabolism functions to provide both energy and C skeletons for plant growth and N assimilation^8,10^. Our results showed that the midrib had lower chlophyll a chlophyll b, carotenoid, and SSA than that of the lamina (Fig. 1i–l). The previous study has shown that the midribs tend to have fewer chloroplasts in C_3_ and CAM plants, which might be the reason for lower chlophyll content in the midrib^24^. More than 30 genes are involved in the chlophyll biosynthesis pathway and any genetic mutation may affect the synthesis of chlorophyll^25^. *HEMA1* is considered to play the major role in tetrapyrrole biosynthesis and antisense *HEMA1* *Arabidopsis* plants showed decreased levels of chlophyll^26^. In *Arabidopsis thaliana,* Alexey et al.^27^ showed that the chlorophyll biosynthesis pathway was suppressed in this *ChlI* mutant. In accordance with this, our results found that some key genes related to pigment biosynthetic process and C metabolism were significantly down-regulated in the midrib, including *LOC107777980 (MgPME)*^28^, *LOC107783891 (CHLI)*^29^, *LOC107763283 (hemA)*, and *LOC107783257 (CHLP)*^30^, which play crucial roles in chlophyll biosynthesis, *LOC107772713 (PSY2)*^31,32^, which encodes phytoene synthase and controls the carbon flux through the carotenoid biosynthetic pathway, *LOC107785687 (SPS2)* edcoding sucrose-phosphate synthase that plays the role of rate-limiting steps in sucrose synthesis in higher plant^33^. The down-regulation of these genes might decrease the chlorophyll formation and photosynthesis efficiency in the midrib. Besides, the lower chlorophyll content resulted in a decrease of the chlorophyll a/b binding proteins in midrib. *LOC107772663 (LHCb1)*, which is one of the most abundant chloroplast proteins in plants and mainly functions to collect and transfer light energy to photosynthetic reaction centers^34^, was significantly repressed in midrib. Previous studies showed that that in CAM plants the photochemical parameters describing the performance of PSII were significantly lower in the midribs than in the interveinal leaf area, which reduced the photosynthesis^24^. In the present study, PSI, PSII, and photosynthetic electron transport are key components in the photosynthetic pathway. While *LOC107810205 (PsbR)*, *LOC107784985 (PsaO)* and *LOC107803171 (petF)*, which were involved in PSI, PSII, and photosynthetic electron transport were down regulated in midrib. *LOC107771723 (rbcS)*, which encodes a key enzyme in the calvin cycle and assimilates atmospheric CO_2_ into the biosphere^35^, was also down regulated in midrib. This is consistent with the physiological differences between lamina and midrib. Carbon metabolism is closely related to nitrogen metabolism. The lower capacity of photosynthesis and carbon fixation might influence the nitrogen metabolism and resulted in higher level nitrate in the midrib.

NR and GS are two of the most important enzymes in N assimilation^9^. The ammonium taken up by AMTs or derived from nitrate is used to produce a variety of amino acids via the GS/GOGAT cycle^8^. Lu et al.^36^ showed that expression of a constitutively activated nitrate reductase (NR) enzyme dramatically decreases leaf nitrate levels in burley tobacco. Meanwhile, recent literature also suggests that the overexpression of GS is able to increase the activity of GS and promote N assimilation efficiency^37^. *NLP7* is a primary regulator in nitrate response and regulates the expression of several nitrate-responsive genes including *NIA1*, *NIA2*, *NRT2.1*, and *NRT2.2*^19,20^. And OsNLP4 transactivats the NRE motif at the promoter of OsNiR encoding nitrite reductase in rice^21^. Xiang et al.^38^ has demonstrated that *NLP7*-overexpressing plants showed lower nitrate accumulation. In this study, *NLP7* and *NLP4* were down-regulated in the midrib, which was inconducive to the decrease of nitrate accumulation in the midrib. Further investigation of the expression of genes encoding nitrate response, transport, and assimilation led to the discovery of nitrate response genes (*NPF6.3*, *NLP4*, and *NLP7*), nitrate transporters (*NPF2.13*, *NPF3.1*, *NPF7.3*, *NPF1.2*, and *NPF7.2*), and nitrate assimilation genes (*NIA*, *GS* and *GOGAT*) with contrasting transcriptional responses in lamina and midrib. And our results showed that midrib was lower in NR activity, GS activity, NH_4_-N, and soluble protein content while higher in NO_3_-N and NO_3_-N/TN than midrib, suggesting that midrib might retain a weaker capacity of nitrate assimilation. In plants, NO_3_^−^ accumulation depends on its absorption, transport, and metabolism, among which there is a close interdependency that facilitates the coordinated regulation of NO_3_^−^ accumulation in plants. *NPF7.3/NRT1.5* mediates efflux of NO_3_^–^ to the xylem vessels, whereas *NPF7.2/NRT1.8* performs the opposite function and retrieves NO3–from the xylem sap into xylem parenchyma cells^15,39^. *NPF2.13* can facilitate outward nitrate transport by phloem loading. Moreover, *NPF1.2* is expressed in the companion cells of the major veins in expanded leaves and involved in diverting root-derived nitrate into phloem in the major vein of mature and expanded leaves^40^. qRT-PCR results showed that the nitrate transporter genes *LOC107782967 (NLP4)*, *LOC107806749 (NLP7)*, *LOC107775674 (NPF2.13)*, *LOC107829632 (NPF3.1)*, *LOC107799198 (NPF6.3)*, *LOC107768465 (NPF7.3)* were down-regulated while *LOC107789301 (NPF1.2)* and *LOC107770138 (NPF7.2)* were up-regulated in midrib but not in the lamina, indicating that midrib had poor ability in reallocation nitrate transported by roots.

In Conclusion, significant differences were observed in nitrate accumulation between lamina and midrib of flue-cured tobacco. Pigment content and SSA in midrib were significantly lower than that in the lamina, which resulted in insufficient C skeleton for nitrogen metabolism. Meanwhile, the greater nitrate accumulation was probably conferred by more disadvantageous aspects such as weak nitrogen reduction, weak nitrogen assimilation, poor ability in reallocation, and high capacity of accumulating nitrate in midrib than in the lamina. The above insights to the physiological and molecular basis of carbon and nitrogen differences in lamina and midrib would be helpful for providing direction for decreasing nitrate accumulation in the midrib.

## Materials and methods

### Plant material and study design

The flue-cured tobacco variety K326 was used in this study. Seeds were sterilized with 2% (v/v) sodium hypochlorite for 5 min twice and then were sown in a floating system. Forty days after sowing, seedlings were transplanted in 7.1 cm × 7.8 cm (diameter × depth) plastic pots and cultivated with Hoagland solution. Pot experiments were conducted on substrate culture in the greenhouse that maintained a temperature of 25 ± 2 °C, an average photosynthetic photon flux density of 400 μmol m^−2^ s^−1^, and relative humidity of 80%. Laminas and midribs were collected separately 15 days after seedlings being transplanted. Fully expanded leaves (length > 5 cm, up to down, the fourth leaf from top) from the same position in three pots of each treatment was sampled in an ice box. Half of the samples were frozen in liquid nitrogen and stored in a freezer at − 80 °C, while the other half were deactivated at 105 °C for 20 min and then dried at 60 °C for 48 h. Frozen samples were used for transcriptome analysis, enzyme activity determination, soluble protein and NH_4_-N content investigation. Dried samples were used for determination of nitrate content. Every treatment had three biological replicates. The K326 seeds used in this study were provided by Yunnan Tobacco Company and the collection of the plant material complied with relevant institutional, national and international guidelines and legislation. In preliminary tests, laminas and midribs of seedlings were collected on the 7th, 15th, and 21st days after seedlings being transplanted to determine the difference in nitrate content. The results showed that the nitrate content of midrib was significantly higher than that of the lamina on the 15th day. So laminas and midribs were collected separately 15 days after seedlings being transplanted.

### Assays of nitrate reductase activity (NRA), sucrose synthetase activity (SSA), and glutamine synthetase activity (GSA)

Frozen samples were powdered with liquid N_2_. The activities of SS, NR, and GS were determined using SS, NR, and GS microdetermination kits (Suzhou Comin Biotechnology Co., Ltd, Jiangsu, China), respectively.

### Measurement of pigment content, nitrate, soluble protein, and NH_4_-N content

Nitrate content was determined by the method described in Cataldo^41^. Samples were frozen in liquid N_2_ and used to investigate the pigment content and soluble protein content according to Zou^42^. About 0.5 g of each sample were frozen in liquid N_2_ and used to investigate the NH_4_-N content according to Fan^43^.

### RNA extraction, preparation of cDNA library, and sequencing

Total RNA was extracted using the mirVana miRNA Isolation Kit (Ambion, Waltham, MA, USA) following the manufacturer’s protocol. RNA integrity was evaluated using the Agilent 2100 Bioanalyzer (Agilent Technologies, Santa Clara, CA, USA). The samples with RNA integrity (RIN) ≥ 7 were used for the subsequent analysis. The libraries were constructed using TruSeq Stranded mRNA LTSample Prep Kit (Illumina, San Diego, CA, USA) according to the manufacturer’s instructions. These libraries were then sequenced on the Illumina sequencing platform (HiSeqTM 2500) and 125 bp/150 bp paired-end reads were generated. Quality control was assessed on the remaining reads using the NGS QC Toolkit^44^. After removing low quality date, the clean reads were mapped to the reference genome of N. tabacum (assembly Ntab-K326) (ftp://ftp.solgenomics.net/genomes/Nicotiana_tabacum/assembly/Ntab-K326_AWOJ-SS.fa.gz) using tophat software^45^ (v2.1.0).

### Enrichment analysis of differentially expressed genes (DEGs)

Transcript profiles of RNA-seq data were analyzed by calculating the read fragments per kilobase per million mapped reads (FPKM). The FPKM value of each gene was calculated using cufflinks, and the read counts of each gene were obtained using htseq-count^46,47^. DEGs were identified using the DESeq (2012) functions to estimate size factors and using nbinomTest^48^. A *P.adjust* < 0.05 and |logFC|> 2 were set as the thresholds for significantly differential expression. Gene function was annotated based on databases of NR (NCBI non-redundant protein sequences), KOG (Clusters of Orthologous Groups of proteins)^49^, Swiss-Prot (A manually annotated and reviewed protein sequence database)^50^, KO (KEGG Ortholog database)^51^, GO (Gene Ontology)^52^. GO enrichment and KEGG pathway enrichment analyses of the DEGs were conducted using R package GOstats (version: 2.40.0, http://bioconductor.org/packages/release/bioc/html/GOstats.html)^53^.

### Gene expression analysis by qRT-PCR

Expression of eight genes related to nitrogen metabolism was observed. qRT-PCR was performed using Light Real-time PCR Instrument (7900HT FAST, ABI). Reactions were incubated in a 384-well optical plate (Roche, Basel, Swiss) at 50.0 °C for 2 min, 95 °C for 10 min, followed by 40 cycles of 95 °C for 15 s, 60 °C for 60 s. TKF and TKR were used as the endogenous control (Table 2). The expression levels of mRNAs were normalized and calculated using the 2^−ΔΔCt^ method^54^.

**Table 2:** Statistics of sequencing data quality.

Sample	Raw reads	Clean reads	Total reads	Total mapped	Q20 (%)	Q30 (%)	GC content (%)
K326YP_1	48,864,160	48,408,636	48,408,636	45,265,003 (93.51%)	96.87	91.7	43.74
K326YP_2	47,517,992	47,096,404	47,096,404	43,990,986 (93.41%)	96.81	91.6	44.09
K326YP_3	52,667,494	52,209,758	52,209,758	48,818,506 (93.5%)	96.87	91.73	43.97
K326ZM_1	46,260,746	45,909,440	45,909,440	43,168,725 (94.03%)	96.96	91.89	43.16
K326ZM_2	48,407,794	48,000,324	48,000,324	45,097,971 (93.95%)	96.85	91.66	43.24
K326ZM_3	50,465,696	50,053,210	50,053,210	47,136,723 (94.17%)	97.03	92.03	43.27

### Statistical analysis

The figures were processed using GraphPad Prism (v. 8.0.1, GraphPad Software Inc., CA, USA) and correlation analysis and variance between treatments were all processed using SPSS 20.0 (IBM, Palo Alto, CA, USA). For comparison between two data sets, a Student’s t test was used. *P < 0.05, **P < 0.01 were considered statistically significant. All presented data is the mean of three biological replicates (n = 3).

## Data Availability

The sequencing data were deposited in the National Center of Biotechnology Information database (https://www.ncbi.nlm.nih.gov/bioproject/PRJNA720776). The datasets used and/or analyzed during the current study are available from the corresponding author on reasonable request.
